# Midland Hospitals and the Insurance Act

**Published:** 1912-05-18

**Authors:** 


### Midland Hospitals and the Insurance Act.

At a Conference of the Representatives of Midland Voluntary Hospitals, which was held at the General Hospital, Birmingham, on Wednesday last week, the following resolution was unanimously adopted :—

That in the opinion of this Conference it is desirable that as soon as the benefits of the Insurance Act come into operation, and provided that arrangements are made with the medical profession for adequate medical attendance outside the hospitals—

(a) Insured persons applying for treatment in the out-patient departments of voluntary hospitals

(1) Should receive "first-aid" only when they meet with accidents or are in such acute medical or surgical conditions as require immediate help and are unable to obtain access to their own doctors, or in the case of a ticket hospital when they come to the hospital with an out-patient note.

(2) Should receive treatment only when the treatment be of such a special nature that it cannot be carried out elsewhere than at the hospital.

(3) Should obtain a second opinion only on the recommendation of a medical officer on the panel.

(b) In the case of insured persons admitted as in-patients a claim for subscriptions or donations should be made on behalf of the hospital to the approved society of the local Insurance Committee, except where, in the case of a ticket hospital (should the management so decide) an in-patient ticket, such as may be required by the regulations of the hospital, has been produced.

Mr. Neville Chamberlain was in the chair, and fifty-nine delegates were present. Not only the Birmingham hospitals, but institutions of Wolverhampton, Coventry, Warneford, Rugby, Stratford-on-Avon, Shrewsbury, and other Midland towns were represented.

